# Total saponins from *Rubus parvifolius* L. inhibits cell proliferation, migration and invasion of malignant melanoma *in vitro* and *in vivo*

**DOI:** 10.1042/BSR20201178

**Published:** 2021-01-22

**Authors:** Jinfeng Cao, Xue Zhao, Yan Ma, Jian Yang, Fuqiang Li

**Affiliations:** 1 Ophthalmology Department, The Second Hospital of Jilin University, Changchun 130041, China; 2 Otolaryngology Department, The Second Hospital of Jilin University, Changchun 130041, China

**Keywords:** Malignant melanoma, Metastasis, Rubus parvifolius, Total saponins

## Abstract

**Background:** Total saponins from *Rubus parvifolius* L. (TSRP) are the main bioactive fractions responsible for the anti-tumor activities. The work was aimed to evaluate the anti-tumor effect of TSRP in malignant melanoma (MM) *in vitro* and *in vivo*.

**Methods and results:** Anti-melanoma cell proliferation, invasion and migration effect of TSRP were detected in human MM A375 cells under the indicated time and dosages. *In vivo* anti-tumor effect of TSRP was measured in A375 xenograft immunodeficient nude mice. Sixty A375 xenografts were randomly divided into five groups: Vehicle, cyclophosphamide (CTX, 20 mg/kg), TSRP (25 mg/kg), TSRP (50 mg/kg) and TSRP (100 mg/kg) groups for 14 days’ treatment. In addition, the melanoma metastasis in lung *in vivo* of TSRP was detected in A375 tail vein injection mice, and the histopathalogical analysis of the lung metastasis was detected by Hematoxylin–Eosin (H&E) staining. TSRP significantly inhibited the cell proliferation, invasion and migration of A375 *in vitro* at the indicated time and dosages. TSRP treatment effectively blocked the tumor growth in immunodeficient nude mice. In addition, TSRP also significantly inhibited the lung metastasis of melanoma.

**Conclusion:** The present study indicated that the TSRP has a remarkable anti-MM effect, which mainly through the inhibition of the cell invasion, migration and tumor metastasis.

## Introduction

Malignant melanoma (MM) is a highly aggressive and highly malignant tumor. Most of the MMs originate from the skin, and also from the eyes and nasal cavity [1–3]. Melanoma is the fifth most common malignant tumor in men and the sixth most common in women worldwide. The incidence of melanoma has increased year by year in recent times. While the hidden early symptoms of melanoma lead to a low rate of early diagnosis, once diagnosed, most melanomas reach the advanced stage, which lead to a persistent high mortality rate of melanoma [4,5]. Rapid progress, poor prognosis and high mortality are the characteristics of MM [6]. The mortality of cutaneous malignant melanoma (CMM) accounts for 80% of skin-related tumors, which has been high for many years [3,7]. Metastasis can occur in early stage of melanoma; lung and brain are the most common sites of metastasis [8,9]. At present, the main therapies of melanoma are surgical excision, adjuvant radiotherapy and chemotherapy. But because of the insensitivity of melanoma to radiotherapy and chemotherapy, the therapeutic effect is not good [10,11]. The high mortality rate of melanoma has seriously endangered the health of people all over the world. Thus, searching for low toxicity and high efficiency anti-cancer drugs is an important direction in the field of melanoma treatment. Recent studies have found that traditional Chinese medicine plays an important role in cancer treatment, such as *Salvia miltiorrhiza, Ligusticum chuanxiong* or curcumin, which provided a new strategy for improving the prognosis and therapy of melanoma.

*Rubus parvifolius* L. (RP), also named Tianqingdi Baicao, Hongmeixiao and March Bubble, is a dry root plant of *Rubus* L., Rosaceae [12]. RP has a long history of application in the folk, according to the records of medical ancient book, ‘Ben Cao Shi Yi.’ It is mainly used to treat hematemesis, knife injury, postpartum stasis abdominal pain, dysentery, hemorrhoids, hemorrhoids and jaundice. In addition, the root of RP is also used for the treatment of menorrhagia in women [13]. Previous studies on RP mainly focused on the pharmacological activity of heart and brain organs, but in recent years, the anti-cancer research of RP has gradually increased. The chemical constituents’ research of RP have been reported that RP mainly contains triterpenoids, triterpenoid saponins and flavonoids, which possess the activity of anti-inflammation, anti-oxidation and anti-tumor *in vitro* [14–17]. Furthermore, it was reported that the water extract and total saponins of RP (TSRP) exhibit the protective effect on focal cerebral ischemia [18], and the anti-tumor effect on chronic myeloid leukemia *in vitro* and xenografts [12,19–21]. In an *in vitro* experiment, Seleshe et al. showed that RP has the antioxidant and antimicrobial effects [17]. The study of Cai et al. exhibited that RP showed an antibacterial effect *in vitro* [22]. In *in vivo* animal experiment, Zhang et al. reported that RP can inhibit the growth of leukemia K562 cells *in vitro* and *in vivo* animals [12]. Xu et al. reported that the TSRP exhibited anti-leukemia effect *in vivo* through STAT3 and eIF4E signaling pathway [20]. In addition, the hepatoprotective and antioxidant activities of RP were reported by Gao et al. in *in vivo* animals [13], and the neuroprotective effects of TSRP on cerebral ischemia/reperfusion injury in rats was studied by Wang et al. [18]. However, there is no research study reported for the effect of RP on MM *in vitro* and *in vivo*.

In the present study, we extracted the TSRP, and studied its cell anti-proliferative activity, cell invasion and cell migration activity in human MM cell A375 *in vitro*. Furthermore, we also studied the anti-tumor and lung metastasis effect of TSRP in A375 xenografts and A375 tail vein injection mice *in vivo*. The results exhibited that TSRP showed a good anti-tumor and anti-metastasis effects on MM cells *in vitro* and *in vivo*.

## Materials and methods

### Materials and reagents

Human MM cell line A375 was obtained from ATCC (Manassas, U.S.A.). 3-(4,5-dimethylthiazol-2-yl)-5-(3-carboxymethoxyphenyl)-2-(4-sulfophenyl)-2H-tetrazolium (MTS) was purchased from Promega (Madison, U.S.A.). Dulbecco’s Modified Eagle’s Medium (DMEM) and fetal bovine serum (FBS) were obtained from Gibco (Waltham, USA). Cyclophosphamide (CTX) was purchased from Sigma (St. Louis, U.S.A.).

### Preparation of TSRP

TSRP was provided by the Ophthalmology Department of the Second Hospital of Jilin University. The products of TSRP were identified by Dr. Fuqiang Li (Ophthalmology Department, The Second Hospital of Jilin University), and a voucher specimen of TSRP had been deposited in the herbarium of Jilin University with the deposition number: RP-201811. The process of the preparation is as follows [19]: the plant materials were crushed, filtered through 40-mesh sieve, extracted with 70% ethanol four times, combined the filtrates, and concentrated the filtrates to no ethanol residue. Water was added to the concentrates with the ratio of 1:1, then loaded to D101 resin column, washed with water for 5 column volume. Then, elution with 70% ethanol for 6 column volume was performed, the eluent was collected, ethanol was recovered and concentrated to a thick paste, dried in vacuum and crushed. The yield was 1.65%.

### Cell culture

Human MM cell line A375 were cultured with the cell culture medium of DMEM containing 10% FBS and 1% penicillin–streptomycin. The cells were cultured for 2–3 days in the humidified incubator at 37°C and 5% CO_2_. The logarithmic phase cells were digested with 0.25% trypsin, the cells were collected, and then, the concentration of the cells was adjusted to 1 × 10^7^/ml for *in vivo* xenograft use.

### Anti-proliferative activity assay *in vitro*

The anti-proliferative activity of TSRP in human A375 cells was measured by MTS method. The cells in logarithmic growth phase of A375 were planted in a 96-well plate at a concentration of 4 × 10^3^/ml, 100 µl per well. The cells were incubated for 12 h in the cell culture incubator at 37°C before drug treatment. The different concentrations of drug (0.1, 0.3, 1, 3, 10, 30, 100, 300 µg/ml) and vehicle solution (DMSO) were diluted with complete cell culture medium (the stock drug concentration was 100×), and the medium in 96-well plate was replaced. Then the cells with drug were continued to be incubated at 37°C for 24, 48 and 72 h, respectively.

At the indicated times, 20 μl MTS solution was added to each well. The cells were continued to incubate at 37°C for 2 h. Then, the plates were shaken for 10 min on a plateshaker by slowly increasing the shaking speed to a maximum of 700 shakes/min; then the absorbance of the plate was read at a wavelength of 492 nm by a microplate reader. The relative cell viability was calculated with the formula: Relative cell viability % = Absorbance value of drug/Absorbance value of vehicle × 100. Each test concentration was performed in triplicates and was repeated three times.

### Cell invasion analysis *in vitro*

The logarithmic phase of human A375 cells with 0.25% trypsin was digested and collected. Then, the suspended cells with the invasion medium of DMEM + 0.1% BSA was diluted to 1 × 10^5^/ml. A total of 100 µl of A375 cells were taken to add on the top of the transwell membrane in the upper chamber. And 600 µl of DMEM + 10% FBS were added with the related drugs to the lower chamber in 24-well plate, and incubated for 48 h. Finally the cells were stained with 0.4% Trypan Blue and imaged.

### Cell migration analysis *in vitro*

The logarithmic phase of human A375 cells with 0.25% trypsin were digested and collected. Then, the cells were diluted to the concentration of 1 × 10^5^/ml. A 1-ml cell suspension was seeded to each well of 24-well plate. After the cells were attached and the cell density reached ∼60%, 20 μl of pipette tips were used to make a line. Then fresh medium with the related drugs was changed and imaged with microscope as the initial width. After 48 h drug treatment, the cells were imaged again, and calculated the migration distance.

### Animals

Male BALB/C nude mice (body weight (BW): 20 ± 2 g) were provided by the animal experimental center of Wish Technology Ltd. (Changchun, China). All the mice were reared in the animal experimental center of Wish Technology Ltd. In SPF grade environment with free access to food and water (24 ± 1°C, 50 ± 5% of humidity and 12-h day/night cycle). Six mice were raised in one polyacrylic cage, the mice received humane care on the lines of National Institutes of Health Guidelines of the U.S.A. (National Research Council of U.S.A., 1996) and the ethical regulations of Wish Technology Ltd. with the Protocol Number: SMP-IACUC-002-F-01. All the mice were quarantined for 1 week before the use.

### Anti-tumor experiment *in vivo*

The human MM cell line A375 was inoculated into the left axilla in BALB/C nude mice (1 × 10^6^/mice). The xenografts were randomly divided into five groups (Vehicle group, CTX 20 mg/kg group, TSRP 25, 50 and 100 mg/kg groups, *n*=12), when the tumor size grew to 100 mm^3^, after 10 days of inoculation. All the mice were injected with the related drugs in the volume of 10 ml/kg/day BW by intraperitoneal injection, respectively. The tumor size and BW were measured every 3 days. At the end of the experiment (treatment for 14 days), all the mice were killed by cervical dislocation. The tumor tissues were harvested and stored in liquid nitrogen for later use.

### Lung metastasis experiment *in vivo*

The logarithmic growth of A375 cells with 0.25% trypsin was collected and digested. Then, the normal saline was used to digest the cells to 5 × 10^6^/ml. A total of 200 μl tumor cell suspension was injected into BALB/C nude mice through tail vein (1 × 10^6^/mice). Related drugs (Vehicle group, CTX 20 mg/kg group, TSRP 25, 50 and 100 mg/kg groups, *n*=12) were admistered after the second day of inoculation with the volume of 10 ml/kg/day BW by intraperitoneal injection for 14 days treatment. After the end of the experiment, the mice were killed by cervical dislocation. The lung tissues were harvested, imaged and underwent histopathological analysis.

### Histopathological analysis

The lung specimens were fixed with 40 g/l formaldehyde solution overnight. Then, the fixed specimens were embedded in paraffin, cut into 5-µm-thick sections and stained with Hematoxylin–Eosin (H&E) in terms of the routine histopathological examination. The final stained sections were photographed under a light microscope (BX-50 Olympus) at 200× magnification.

### HPLC analysis for TSRP

The chromatographic column: Merck Chromolith® Flash (2 mm × 25 mm) in tandem with Merck Chromolith® Flash (2 mm × 50 mm). Mobile phase: 2.5 mmol/l ammonium acetate (w/v), 0.05% ammonia aqueous solution (v/v) (A) - acetonitrile (C) and methanol (D). Gradient elution: 0–5 min, 10% C; 5–7 min, 10–15% C; 7–15 min, 15–40% C; 15–40 min, 40–50% C, 0–20% D; 40–45 min, 50% C, 20–33.4% D; 45–50 min, 50–10% C, 33.4–90% D; 55–60 min, 10% C, 90% D; flow rate: 0.2 ml/min. Column temperature: 45°C. Injection volume: 2 μl.

### Statistical analysis

All the values were represented as mean ± SD. The statistical comparisons were calculated by means of a one-way ANOVA test followed by Dunett’s *t* test with SPSS19.0 and GraphPad Prism 6.0 statistical software. *P*<0.05 and <0.01 were regarded as statistically significant.

## Results

### Anti-proliferative activity of TSRP on human A375 cell line *in vitro*

**Figure 1:**
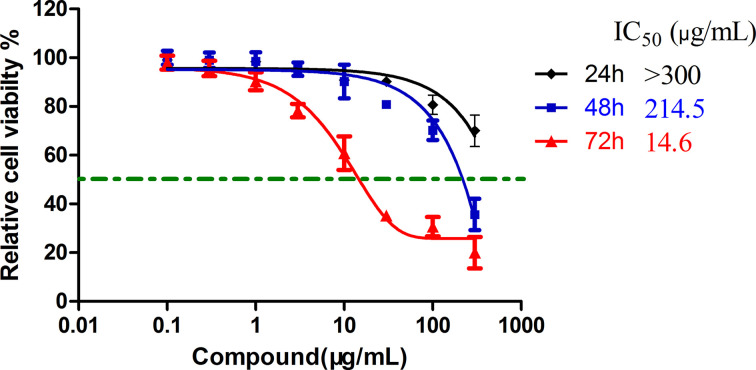
The cell anti-proliferative activity of TSRP in human A375 cells *in vitro* The values were collected by three independent experiments.

First, we detected the anti-proliferative activity of TSRP on human A375 cells *in vitro* in order to evaluate the anti-tumor effect of TSRP. As shown in Figure 1, we detected the cell anti-proliferative activity in the concentration of 0.1, 0.3, 1, 3, 10, 30, 100 and 300 µg/ml for the treatment time of 24, 48 and 72 h, respectively. The result showed that TSRP significantly decreased the cell viability in 72 h with the half inhibition concentration (IC_50_) of 14.6 µg/ml. While the IC_50_ for TSRP was more than 300 µg/ml in 24 h and 214.5 µg/ml in 48 h, the result exhibited that longer the treatment time higher the anti-proliferative effect of TSRP.

### The cell invasion effect of TSRP on human A375 cell line *in vitro*

**Figure 2:**
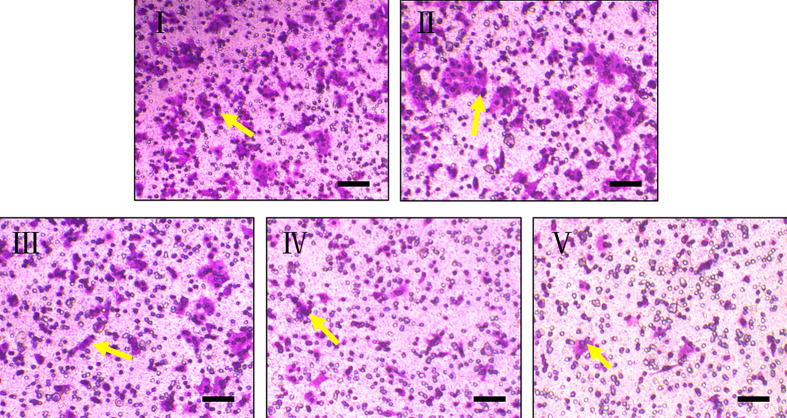
The effect of TSRP on cell invasion of human A375 cells *in vitro* Representative photographs of cell invasion through transwell experiment (scale bar: 200 μm). I, Vehicle group; II, CTX 150 μg/ml group; III, TSRP 30 μg/ml group; IV, TSRP 60 μg/ml group; V, TSRP 120 μg/ml group. The arrowheads indicate the invasion cells.

In addition to the unlimited proliferation potential of MM tumors, invasive growth is also an important feature of MM tumors, which is different from benign tumors. Thus, in order to detect the effect of TSRP on melanoma cell invasion of A375, we established the transwell experiment model to assay the cell invasion *in vitro* (Figure 2). The result in Figure 2 exhibited that the cells in vehicle group have a strong invasion ability. CTX (150 μg/ml) and TSRP (30, 60 and 120 μg/ml) treated cells dramatically inhibited the cell invasion in different degrees, compared with vehicle group. In the dosage of TSRP, the highest dosage of 120 μg/ml TSRP showed the highest inhibition effect in human A375 cell invasion.

### The cell migration effect of TSRP on human A375 cell line *in vitro*

**Figure 3:**
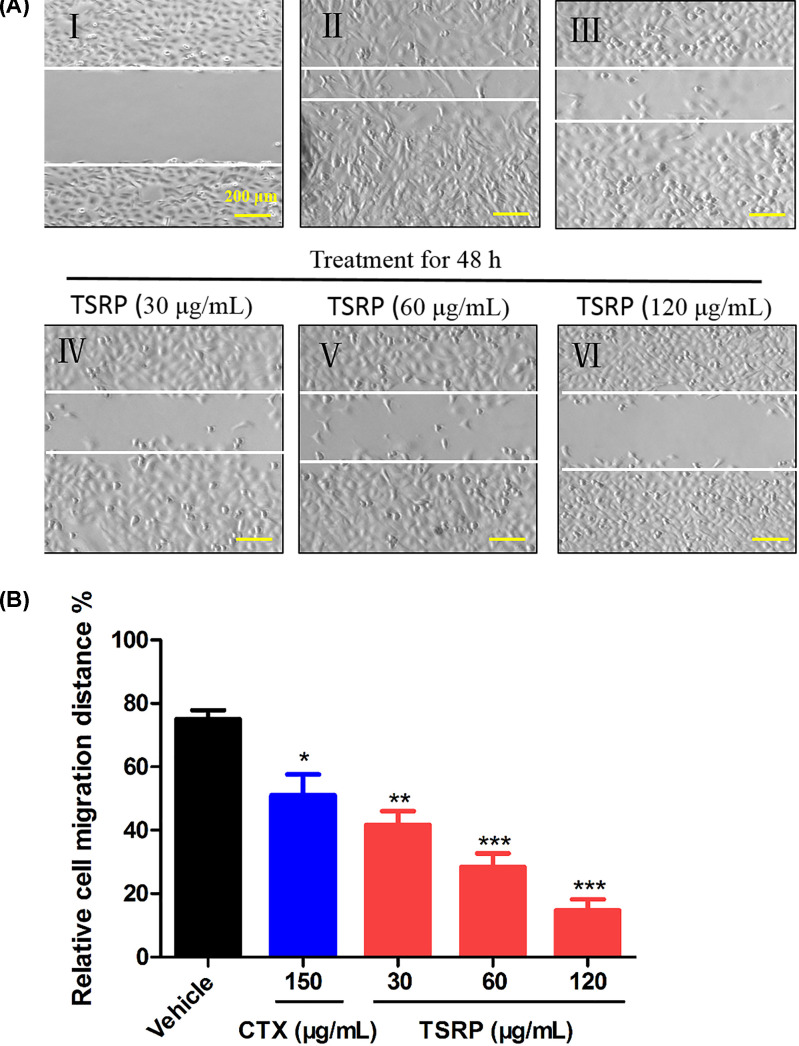
The effect of TSRP on cell migration of human A375 cells *in vitro* (**A**) Representative photographs of cell migration. I, Initial distance of control group before drug treatment. II–VI, Cell migration distance in vehicle group, CTX 150 μg/ml group, TSRP 30 μg/ml group, TSRP 60 μg/ml group and TSRP 120 μg/ml group, respectively, after drug treatment of 48 h. (**B**) Quantification of the cell migration distance. *P<0.05, **P<0.01, ***P<0.001 vs vehicle group.

Furthermore, as we know, MM tumors are easily migrated to the whole body. Herein, we wondered if TSRP could inhibit the melanoma cell migration *in vitro*. The result in Figure 3 showed that after 48-h treatment, the cells in vehicle group have strong migration ability, which are almost migrated to the center and the wound almost heals. While the CTX treated cells have a significant inhibition on the wound healing effect (*P*<0.05, Figure 3), when compared with the vehicle group. TSRP treated cells in the dosage of 30, 60 and 120 μg/ml dramatically decreased the cell migration, and had a strong wound healing effect, especially at high dose of TSRP treated cells, compared with the vehicle group (*P*<0.01, *P*<0.001, Figure 3).

### Anti-tumor effect of TSRP on human A375 xenografts *in vivo*

**Figure 4:**
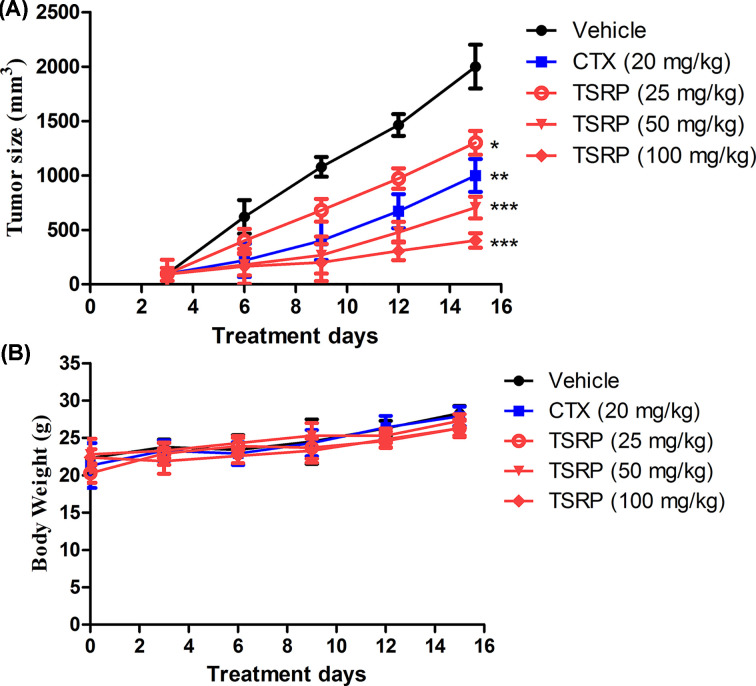
Anti-tumor effect of TSRP on human A375 xenografts *in vivo* (*n*=12) (**A**) Tumor size changes during the whole experiment. (**B**) BW. *P<0.05, **P<0.01, ***P<0.001 vs vehicle group.

Based on the results of the cell proliferation, cell invasion and cell migration in MM *in vitro*, we continued studying the anti-tumor effect of TSRP in human A375 ectopic xenografts through subcutaneous transplantation. As shown in Figure 4, TSRP had a remarkable anti-tumor effect in A375 ectopic xenografts *in vivo* (*P*<0.05, *P*<0.001), compared with the vehicle group. After TSRP treatment, the tumor size were significantly reduced in the dosage of 25, 50 and 100 mg/kg in a dose-dependent manner (Figure 4A, *P*<0.05, *P*<0.001) for continuous 14 days treatment, compared with vehicle group. In addition, in the whole *in vivo* anti-tumor experiment, all the animals were alive. The statistical data of the animal BW showed that treatment with TSRP did not influence the animal BW, even at the high dosage of 100 mg/kg (Figure 4B), which demonstrated that TSRP has a low toxicity *in vivo*.

### Effect of TSRP on human A375 lung metastasis experiment *in vivo*

**Figure 5:**
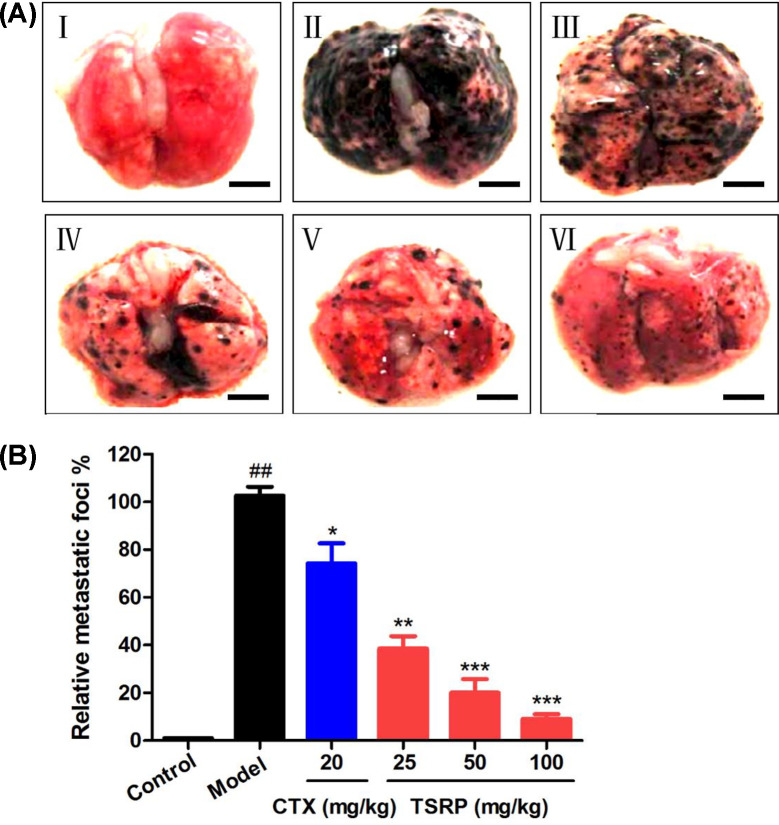
Morphological observation of the metastatic lung in human A375 cell-bearing mice (**A**) Representative lung metastatic images (scale bar: 500 μm). I, Control group; II, Model group; III, CTX 20 mg/kg group; IV, TSRP 25 mg/kg group; V, TSRP 50 mg/kg group; VI, TSRP 100 mg/kg group. (**B**) Quantification of the metastasis in lung. *^##^P*<0.01 vs control group, *P<0.05, **P<0.01, ***P<0.001 vs model group.

Since we noticed the strong effect of TSRP in *in vivo* MM xenografts, we further explored the metastasis effect of TSRP in lung *in vivo*. As shown in Figure 5, we observed that the melanoma cells almost covered the whole lung in the model group (Figure 5II). CTX (20 mg/kg) treated group had a little better metastasis inhibition effect compared with the model group (Figure 5III). While TSRP treated group in the dosage of 25, 50 and 100 mg/kg significantly inhibited the melanoma cell metastasis to lung, especially in the high dose of TSRP (100 mg/kg) treated mice, compared with the model group (Figure 5IV–VI). From this result, we could observe a strong metastasis inhibitory effect of TSRP, which implied that TSRP may be a potential therapeutic strategy for melanoma metastasis.

### Histopathological changes in lung tissue

**Figure 6:**
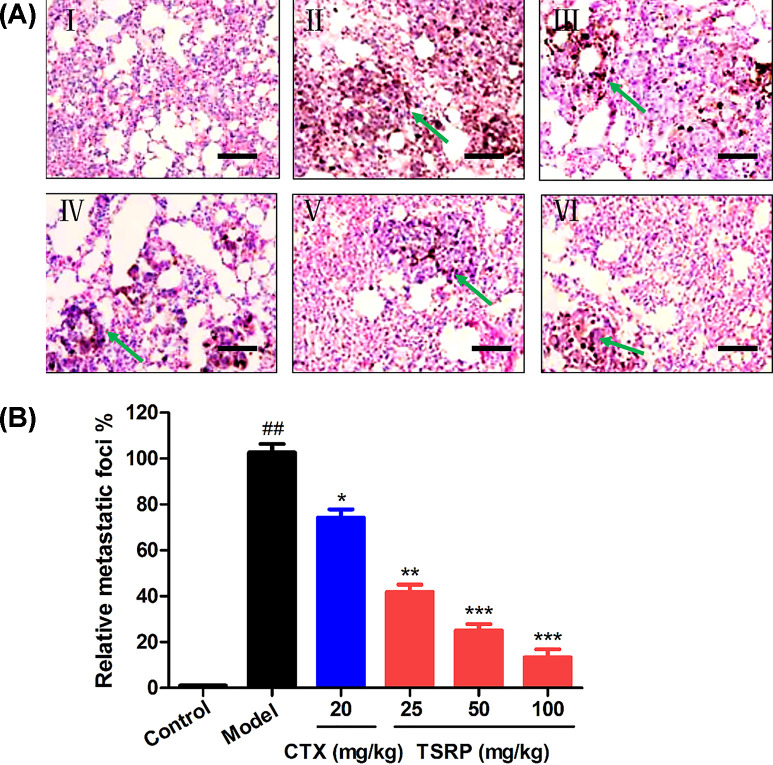
Histopathological examinations of the metastatic lung in human A375 cell-bearing mice (**A**) Representative histopathological images of lung by H&E staining (400×, scale bar: 50 μm). I, Control group; II, Model group; III, CTX 20 mg/kg group; IV, TSRP 25 mg/kg group; V, TSRP 50 mg/kg group; VI, TSRP 100 mg/kg group. The arrowheads indicate the metastatic foci. (**B**) Quantification of the metastatic foci. *^##^P*<0.01 vs control group, *P<0.05, **P<0.01, ***P<0.001 vs model group.

At the same time, in order to avoid the false positive in lung metastasis, we continued detecting the histopathological changes in lung (Figure 6). From Figure 6I, we observed there was no metastasis foci, the lung had an intact architecture in control group under the photomicroscope. While in Figure 6II, the architecture of the lung section was strongly destructed, and there were many foci in the model group (*P*<0.01). Furthermore, consistent with the lung morphology result, we observed that TSRP treated group significantly decreased the metastasis foci in each dosage, compared with that in the model group (Figure 6 IV–VI, *P*<0.01, *P*<0.001).

### HPLC profile of TSRP

**Figure 7:**
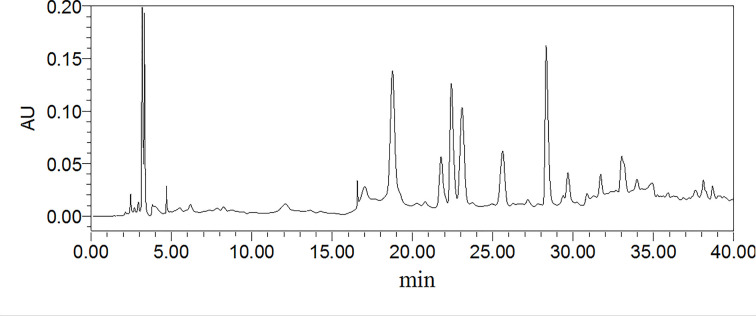
HPLC profile of the TSRP fraction

In order to clarify the bioactive constituents of TSRP fraction, we established the HPLC analysis method for TSRP. From Figure 7, we observed that the chemical constituents of TSRP had a broad heterogeneity, and the high content constituents mainly focused on the peak time of 15–35 min, providing useful information for us to further identify the mainly bioactive constitutes in our future work for TSRP. The data of the bioactive constitutes isolation, identification and the activity identification of TSRP will be reported in due course.

## Discussion

MM originates from nevus cells or melanocytes. It often occurs in skin and mucosa, and can also occur in the eyes, nasal cavity, pharynx and throat, anus and rectum, and the central nervous system [23,24]. CMM is the most common malignant tumor in primary skin tumors and accounts for ∼80% of all skin cancer deaths [25]. MM is characterized by rapid occurrence, prone to distant metastasis and poor prognosis [26]. It is the leading cause of skin cancer deaths in European and American countries with light skin color [27]. The most common primary cutaneous melanoma in Asian population is acral melanoma, which accounts for ∼50% of all melanomas. The upper limbs are mainly at the end of the fingers and under the nails, and the lower limbs are mainly at the toes and soles of the feet. According to statistics, the incidence of melanoma in the world increases by 3–8% every year [28]. Therefore, early diagnosis of advanced or diffuse melanoma is particularly critical. In addition, acidic microenvironment and fat particles can up-regulate interstitial marker proteins, inhibit E-cadherin and other epithelial marker proteins, trigger epithelial–mesenchymal transition, and promote lung metastasis of melanoma cells [29,30], which is one of the main causes of death in MM patients.

In our study, we would like to explore an efficient drug for the therapy of MM. RP is widely distributed in East and South Asia [13]. It is traditionally used as a herbal therapy medicine for fever, angina, dysentery, enteritis, concretion, rheumatism, eczema, dermatitis and so on. Saponins are one of the important components of the natural products. They have a variety of biological activities, such as anti-tumor, anti-inflammation, immunomodulation, antiviral, antifungal, spermicidal and hepatoprotection. From the results of our study, we observed that TSRP significantly inhibited the cell proliferation of human melanoma A375 cells with the IC_50_ of 14.6 μg/ml at 72-h treatment. The TSRP also significantly inhibited the human melanoma A375 cells invasion and migration *in vitro* at the indicated time and dosages. Furthermore, in the following *in vivo* experiment, TSRP exhibited an efficient anti-tumor effect, which significantly blocked the tumor growth in immunodeficient nude mice, with little toxicity observed in the mice. More importantly, we found that TSRP remarkably inhibited the lung metastasis of melanoma, showing a therapeutic potential.

Based on the excellent anti-tumor and anti-metastasis result of TSRP, we will further clarify the mechanism of TSRP targeting melanoma in our future work. According to the previous literature, we found that TSRP could induce cell apoptosis on HL-60 leukemia cells, which is mainly through Bcl-2 and Fas signaling pathway in protein and mRNA levels [19]. In addition, Ge et al. and Xu et al. also reported that the anti-tumor effect of TSRP on HL-60 leukemia cells could inhibit the signaling pathways of STAT3, AMPK and eIF4E [19,20].

In conclusion, the present study indicates that the TSRP has a remarkable anti-MM effect, which is mainly through the inhibition of the cell proliferation, cell invasion and cell migration *in vitro* and *in vivo*. The *in vivo* anti-tumor and anti-metastasis study also showes the safety of TSRP *in vivo*. Therefore, TSRP is expected to become a potential new drug strategy for treating the MM. However, the mechanisms of TSRP against MM remains to be further evaluated.

## Data Availability

All data generated or analyzed during the present study are included in this published article.

## Abbreviations

BW: body weight
CMM: cutaneous malignant melanoma
CTX: cyclophosphamide
DMEM: Dulbecco’s Modified Eagle’s Medium
eIF4E: eukaryotic translation initiation factor 4E
FBS: fetal bovine serum
IC_50_: half inhibition concentration
MM: malignant melanoma
MTS: 3-(4,5-dimethylthiazol-2-yl)-5-(3-carboxymethoxyphenyl)-2-(4-sulfophenyl)-2H-tetrazolium
RP: *Rubus parvifolius* L.
SPF: specific pathogen free
TSRP: total saponins from *Rubus parvifolius* L
